# An Equilibrium State Diagram for Storage Stability and Conservation of Active Ingredients in a Functional Food Based on Polysaccharides Blends

**DOI:** 10.3390/polym15020367

**Published:** 2023-01-10

**Authors:** César Leyva-Porras, Zenaida Saavedra-Leos, Manuel Román-Aguirre, Carlos Arzate-Quintana, Alva R. Castillo-González, Andrés I. González-Jácquez, Fernanda Gómez-Loya

**Affiliations:** 1 Centro de Investigación en Materiales Avanzados S.C. (CIMAV), Miguel de Cervantes No. 120, Complejo Industrial Chihuahua, Chihuahua 31136, Mexico; 2 Coordinación Académica Región Altiplano (COARA), Universidad Autónoma de San Luis Potosí, Matehuala, San Luis Potosi 78700, Mexico; 3 Facultad de Medicina y Ciencias Biomédicas, Universidad Autónoma de Chihuahua (UACH), Circuito Universitario 31109, Campus UACH II, Chihuahua 31125, Mexico

**Keywords:** equilibrium state diagram, functional food, polysaccharide blends, antioxidant activity, bacteria viabililty (*Bacillus clausii*), co-microencapsulation

## Abstract

A functional food as a matrix based on a blend of carbohydrate polymers (25% maltodextrin and 75% inulin) with quercetin and *Bacillus claussi* to supply antioxidant and probiotic properties was prepared by spray drying. The powders were characterized physiochemically, including by moisture adsorption isotherms, X-ray diffraction (XRD), scanning electron microscopy (SEM), and modulated differential scanning calorimetry (MDSC). The type III adsorption isotherm developed at 35 °C presented a monolayer content of 2.79 g of water for every 100 g of dry sample. The microstructure determined by XRD presented three regions identified as amorphous, semicrystalline, and crystalline-rubbery states. SEM micrographs showed variations in the morphology according to the microstructural regions as (i) spherical particles with smooth surfaces, (ii) a mixture of spherical particles and irregular particles with heterogeneous surfaces, and (iii) agglomerated irregular-shape particles. The blend’s functional performance demonstrated antioxidant activities of approximately 50% of DPPH scavenging capacity and viability values of 6.5 Log10 CFU/g. These results demonstrated that the blend displayed functional food behavior over the complete interval of water activities. The equilibrium state diagram was significant for identifying the storage conditions that promote the preservation of functional food properties and those where the collapse of the microstructure occurs.

## 1. Introduction

Carbohydrate polymers are molecules found in nature with comparable properties to synthetic polymers, for instance, glass transition temperature (Tg), melting temperature (Tm), and molecular weight distribution (MWD) [1,2]. Commonly, polysaccharide molecules include glucose, sucrose, dextrose, arabinose, and galactose. In the food and pharmaceutical industries, polysaccharides (e.g., starch, chitosan, maltodextrins (MX), inulin (IN), and gum Arabic (GA)) are used as carrier agents in the conservation and microencapsulation of active ingredients [3]. Clearly, the adequate selection of the carrier agents in the blend can affect the microencapsulation efficiency of the active ingredient [4]. For example, Meena et al. [5] microencapsulated curcumin, employing blends of whey protein (WP), MX, and GA. They found that the content of the active ingredient remained at values of 92% and 90% at storage temperatures of 25 and 35 °C, respectively, for a period of 6 months. These carrier agents were selected to increase the microencapsulation capability of WP. With the addition of GA, the charge interactions between the positively and negatively charged fractions of WP and GA, respectively, increased the microencapsulation capacity [6,7]. Likewise, MX suppresses the hygroscopicity of GA [8]. Haladyn et al. [9] found that the stability of polyphenolic compounds in microspheres after 14 and 28 days of storage was increased by adding guar gum and sodium alginate, while decreasing with the addition of chitosan. Recently, Saavedra-Leos et al. [10] informed about the microencapsulating properties of a functional food based on IN-MX blends. They found that the antioxidant activity (AA) of quercetin improved with the incorporation of IN; conversely, the addition of MX was beneficial for the microorganisms (*Bacillus clausii*) since it promoted their viability in the presence of IN. 

By definition, a functional food is any healthy food consumed in a regular diet that has health-promoting or disease-preventing properties and physiological benefits [11]. A regular consumption of functional foods provides additional health benefits beyond those traditionally associated with nutrients [12], decreasing the risk of suffering chronic diseases [13]. Kaur and Kas [11] classified the functional foods as: (i) fortified food products with high-quality ingredients. (ii) Foods released to respond to anti-nutritional compounds (iii) Improvement of food raw materials by increasing specific components, e.g., the alimentation of animals; (iv) novel foods produced by genetic manipulation or selection of new varieties; (v) probiotics and prebiotics, which are functional foods containing living organisms. Overall, functional foods classified in (i) and (v) commonly include the incorporation of probiotics and antioxidants as active compounds [14,15]. The term "probiotics" refers to live strains of selected microorganisms that confer health benefits on the host after adequate administration in suitable amounts [16]. The benefits of consuming probiotics can vary with the dose, the type of strain, and the diverse components employed in the formulation of the food. On the other hand, antioxidants are another type of bioactive compound with the ability to strengthen the immune system while also delaying cellular aging [17,18]. A natural source of antioxidants is phenolic compounds, which involve a large group of metabolites that exist in plants [19]. These can be classified into two main groups: flavonoids and non-flavonoids, such as quercetin and resveratrol, respectively. Antioxidants interact with unstable free radicals, inhibiting chain reactions that cause cellular aging and further chronic degenerative diseases [20]. Nevertheless, its consumption is limited, first by the high dose necessary to inhibit all oxidative processes within the body and, second, by instability due to external factors such as decomposition with food processing temperature, reactivity with atmospheric oxygen, and degradation upon exposure to ultraviolet light. Microencapsulation is a strategy implemented to extend the shelf life of bioactive ingredients contained in foods [21,22]. This process consists of trapping and keeping the bioactive ingredients within a protective barrier of a material that can withstand higher processing temperatures without degrading. Consequently, spray drying is a process frequently used in the pharmaceutical and food industries for obtaining dry powder products with a low degradation level and great stability. 

A state diagram is a graphic depiction of the behavior of a system in equilibrium or non-equilibrium, subjected to different conditions such as variations in composition, temperature, humidity, and pressure [23,24]. The complexity depends on the number of properties included. In the food area, these diagrams are commonly used to represent the microstructural stability of foods and the preservation of their properties during storage for a period sufficient to reach moisture adsorption equilibrium [25,26]. As a result, state diagrams of pure compounds have been used as model systems for understanding storage behavior and further development for inclusion in complex food systems [27,28]. For instance, stability state diagrams of pure inulin systems differing in the degree of polymerization (DP) showed differences in the microstructural transformation from amorphous to crystalline [29]. A single state change was shown by the highest DP inulin at an activity of water (a_w_) of 0.5. Meanwhile, the lowest DP inulin showed an intermediate semicrystalline state at a_w_ between 0.3–0.5. Adding low-molecular-weight sugars from orange juice also increased system complexity, revealing that the intermediate semicrystalline interval spans the range from 0.2 to 0.53 [24]. With the aid of a state diagram, Roos [30] found the critical storage parameters for lactose to be a water content of 7.8 g of water for every 100 g of dry mass, an a_w_ of 3.8, and a Tg of 23 °C. Exceeding these parameters during storage, dramatic fluctuations in the physical state of lactose may be observed, including time-dependent crystallization and flow properties. Higl et al. [31] plotted the inactivation constant of microorganisms in lactose at different storage temperatures. They found that as long as the samples remain in the glassy state, moisture adsorption is low and independent of storage temperature. However, when passing to the non-glassy state, the adsorption increases rapidly, as does the inactivation of the microorganisms. Lara-Mota et al. [32] reported the state diagram for β-lactose in an extensive a_w_ interval (0.07–0.972) at storage temperatures of 15, 25 and 35 °C. They discovered that the stability is conserved up to an a_w_ value of 0.742, where mutarotation to α-lactose begins. The wide range of stability corresponds to a low adsorbed water content that remained below the monolayer value (0.0219–0.0409 g of water for every g of dry mass).

Evidently, the development of a state diagram is desirable for both the scientific and technological fields because it allows for the prediction of the behavior of physicochemical properties during storage for a compound containing active ingredients, such as a functional food.

Therefore, the goal of this work is to obtain an equilibrium state diagram for a functional food based on a blend of carbohydrate polymers. These carbohydrate polymers were chosen not only for their performance as carrier agents, but also for the properties they provide for the functional food. For example, MX is a sweet-tasting polysaccharide with an energy intake of 4 kcal/g, while IN has a moderately sweet flavor and provides only 1.5 kcal/g [24,33]. Besides, IN is resistant to the pH conditions found in the stomach and duodenum, reaching the small intestine almost undigested, which helps to metabolize some of the intestinal microorganisms, supplying prebiotic activity. For this purpose, blends of MX and IN in a concentration range of 0–100% by weight were first prepared by spray drying. The food was made functional by adding two active ingredients: quercetin for the antioxidant activity and *B. claussi* microorganisms for its performance as a probiotic. Specifically, this product can be described as a powdered functional food with a sweet taste and low calorie intake, as well as prebiotic, probiotic, and antioxidant properties. The powders were characterized physiochemically, and the antioxidant activity of quercetin and the viability of *B. claussi* microorganisms were determined. The blend with the highest antioxidant activity was selected for moisture adsorption tests at 35 °C. From the adsorption isotherm obtained and the subsequent physicochemical characterizations, the equilibrium state diagram was elaborated. 

## 2. Materials and Methods

### 2.1. Materials

Maltodextrin (MX) and inulin (IN) were employed as carrying agents (Ingredion, Mexico City, Mexico). MX was extracted from cornstarch with a dextrose equivalent (DE) of 10, a DP of 2–16 units of glucose, and a molecular weight of 1625 g/mol. IN was derived from agave. The strain was contained in a solution with bacillus bacteria (*Bacillus clausii*, Bc) purchased from Sanofi-Aventis de Mexico (Mexico City, Mexico). Quercetin 3-d-Galactoside (99%) employed as an antioxidant was acquired from Química Farmacéutica Esteroidal (Mexico City, Mexico). Analytical-grade 2,2-diphenyl-1-picrylhydrazyl (DPPH), (±)-6-hydroxy-2,5,7,8-tetramethylchromane-2-carboxylic acid (Trolox), gallic acid, sodium carbonate (Na_2_CO_3_), and Folin–Ciocalteu reagent were purchased from Sigma–Aldrich Chemical Co (Toluca, Mexico). Inorganic salts with a purity ≥ 90% were purchased from PQM Fermont (Monterrey, Mexico) as microenvironments for subjecting the functional food powders to different conditions of moisture: Sodium hydroxide (NaOH), potassium acetate (CH_3_COOK), magnesium chloride (MgCl_2_), potassium carbonate (K_2_CO_3_), magnesium nitrate [Mg(NO_3_)_2_], sodium nitrate (NaNO_3_), potassium chloride (KCl), and potassium sulfate (K_2_SO_4_).

### 2.2. Preparation of Spray-Dried Powders

For the microencapsulation of the functional food, spray drying was employed. Typically, 20 g of the corresponding carrying agent (inulin, maltodextrin, or a mixture), 1 mg of quercetin, 5 mL of the commercial solution with bacteria (equivalent to a concentration of 2 × 10^12^ CFU), and distilled water for a total volume of 100 mL of solution were mixed in the preparation of the feeding solution. Microencapsulation was carried out in a Mini Spray Dryer B290 (BÜCHI, Labortechnik AG, Flawil, Switzerland) at the following conditions: feed temperature of 40 °C, feeding flow of 7 cm^3^/min, hot airflow of 28 m^3^/h, aspiration of 70%, and pressure of 1.5 bar. Based on the conditions reported in [10], an inlet temperature of 210 °C was selected to obtain the highest antioxidant activity. 

The powders, packed in dark, airtight bags made of low-density polyethylene (LDPE), were kept at 4 °C in darkness for one week until their characterization. Samples were labeled as MX-IN:x-y, where MX and IN stand for the carbohydrate polymer employed as a carrying agent in the blend, and *x* and *y* stand for the concentration of each polysaccharide in the blend in percent by weight.

### 2.3. Water Adsorption Isotherm

The static gravimetric method was employed for constructing the equilibrium sorption isotherms [34] at a temperature of 35 °C. Initially, the drying method determined the moisture content after spray drying. The isotherm points were obtained by subjecting the functional food powder (sample identified as MX-IN:25-75) to different conditions of relative humidity in the interval a_w_ of 0.07–0.972. Microenvironments contained 2 g of powder and 100 g of inorganic salt. The systems were equilibrated for 30 days at the storage temperature. The samples were weighed every 24 h until reaching a constant weight with a difference of ± 0.001 g.

After the incubation lapse, water activity (a_w_) was determined with an Aqualab Series 3 Water Activity Meter (Decagon Devices, Inc., Pullman, WA, USA). The water content measured according to the AOAC method, requires drying the sample in an oven at 110 °C for 2 h. Each experiment was performed in triplicate.

### 2.4. Phisicochemical Characterization

The physicochemical characterizations described below were exerted on the powders from sample MX-IN:25-75 stored at different moisture conditions.

#### 2.4.1. Scanning Electron Microscopy (SEM)

Morphological characterization was conducted using a scanning electron microscope (SEM) (JEOL JSM-7401F, Tokyo, Japan) operated at an accelerating voltage of 2 kV. Powder samples were first dispersed on a double-sided copper conductive tape, then covered with a thin layer of gold utilizing sputtering to reduce charging effects (Denton Desk II sputter coater, Denton, TX, USA). For each sample, several images were acquired at different magnifications (500X, 1000X, 2500X, and 5000X).

#### 2.4.2. X-ray Diffraction (XRD)

An x-ray diffraction (XRD) analysis was carried out to characterize the microstructure. A D8 Advance ECO diffractometer (Bruker, Karlsruhe, Germany) equipped with Cu-K radiation (λ = 1.5406 Å) that operated at 45 kV, 40 mA, and a detector in a Bragg-Brentano geometry was used. Scans were performed in the 2θ range of 5–50°, with step size of 0.016° and 20 s per step.

#### 2.4.3. Thermal Analysis

For determining the glass transition temperature (Tg), a modulated differential scanning calorimeter (MDSC) Q200 (TA Instruments, New Castle, DE, USA) equipped with an RCS90 cooling system was employed. The instrument was calibrated with indium for melting temperature and enthalpy and sapphire for heat capacity (Cp). About 10 mg of the sample were encapsulated in Tzero^®^ aluminum pans. Thermograms were acquired in the temperature range of −50 to 250 °C with a modulation period of 40 s and amplitude of 1.5 °C. These analyses were done in duplicate.

### 2.5. Antioxidant Activity (AA)

The antioxidant activity of phenolic compounds is usually determined by a rapid assay [35]. The most popular tests are based on photometric assays for evaluating the antioxidant activity with reagents such as 2,2’-azino-bis(3-ethylbenzothiazoline-6-sulfonic acid) (ABTS), 2,2-diphenyl-1-picrylhydrazyl (DPPH), oxygen radical absorbance capacity (ORAC), and Folin-Ciocalteu (FC). For the specific case of determining the antioxidant activity of quercetin 3-D-galactose, the ABTS and DPPH assays are very similar.

The extract samples were measured in terms of hydrogen-donating or radical-scavenging activity using the stable DPPH radical. Briefly, 1.7 mL of an alcoholic solution of DPPH (0.1 mmol DPPH/L) were mixed with 1.7 mL of microencapsulated suspension, whose concentration was 30 µg/mL. The mixture was left to stand in darkness for 30 min, and the absorbance at 537 nm was measured using a spectrophotometer, the UV-Vis Evolution 220 (Thermo Scientific, Walthman, MA, USA). The measurements were done in duplicate after the spray-drying process or from storage at different moisture conditions.

### 2.6. Viability of Bacillus Clausii in the Microencapsulated

The number of available Bc bacteria cells was evaluated by means of the plate extension technique with Trypticase-Soy Agar (TSA) (Beckton Dickinson, Germany), using serial dilutions of the encapsulated samples from 1 × 10^−1^ to 1 × 10^−7^. Aerobic growing conditions and an incubation period of 48 h at 37 °C were applied in a Novatech incubator (Guadalajara, Jalisco, Mexico). For the determination of the number of colony-forming units per gram (CFU/g), the concentrations exhibiting between 300 and 30 CFU (1 × 10^−4^ and 1 × 10^−5^) were selected. For the quantification of cultivability, Equation (1) was used. All experiments were repeated five times, and the reported values represent the average of the calculated values.

(1) $$\frac{CFU}{g}=\left[\frac{N{}^{\circ}\;plate\;colonies\times dilution\;factor}{mL\;sample\;seeded}\right]$$

### 2.7. Statistical Analysis

The differences between the mean values of antioxidant activity and viability measurements were determined by one-way analysis of variance (ANOVA). For this purpose, Origin software version 8.5 (OriginLab, Northampton, MA, USA) was employed. A significance value of 0.05 was used in all the calculations.

## 3. Results

### 3.1. Antioxidant Activity of Functional Food Blends

**Figure 1:**
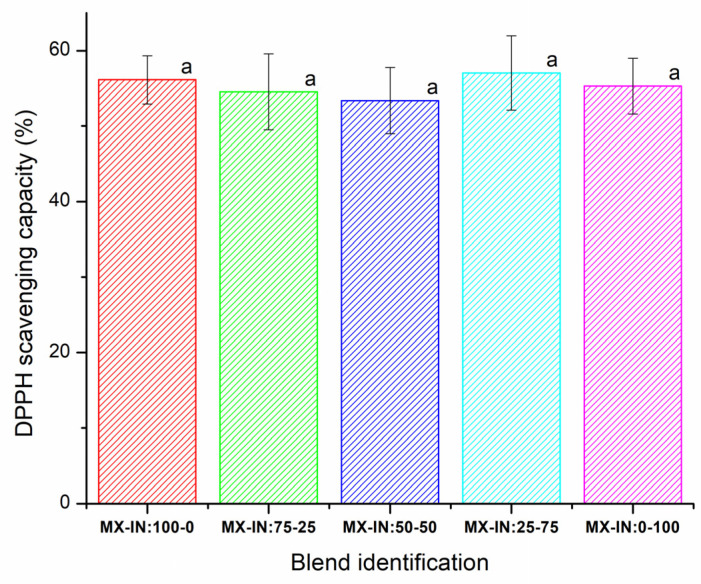
Antioxidant activity of the functional food in all the range of MX-IN concentrations. The lowercase letters indicate mean differences at 0.05 of significance value.

MX-IN concentrations ranging from 0 to 100% were obtained in the prepared spray-dried functional food. In all cases, fine powder materials were obtained, well dispersed, and without signs of microstructural collapse. The physicochemical characterization of these mixtures is beyond the objectives of this work, and only the results of the AA are presented herein. This property was considered suitable to indicate whether the antioxidant ingredient was actually preserved during the spray-drying microencapsulation process. In Figure 1 are shown the results of the AA for the functional food in the entire range of MX-IN concentrations tested. For all the blends, the AA values varied in the range of 53–57%; the one-way ANOVA showed that the population means were not significantly different at the 0.05 level. Although statistically the AA values may be similar, the blend MX-IN:25-75 showed a relatively higher average value (57.03%). Likewise, Saavedra-Leos et al. [10] reported a synergistic effect upon the mixing of MX and IN and found that the MX-IN 1:1 blend presented higher AA values than the pure carrying agents. This agrees with the results presented herein, where the addition of inulin is beneficial for quercetin co-microencapsulation because it promotes higher antioxidant activity.

Based on these observations, sample MX-IN:25-75 was selected for storage under different moisture conditions.

### 3.2. Adsorption Isotherm

**Figure 2:**
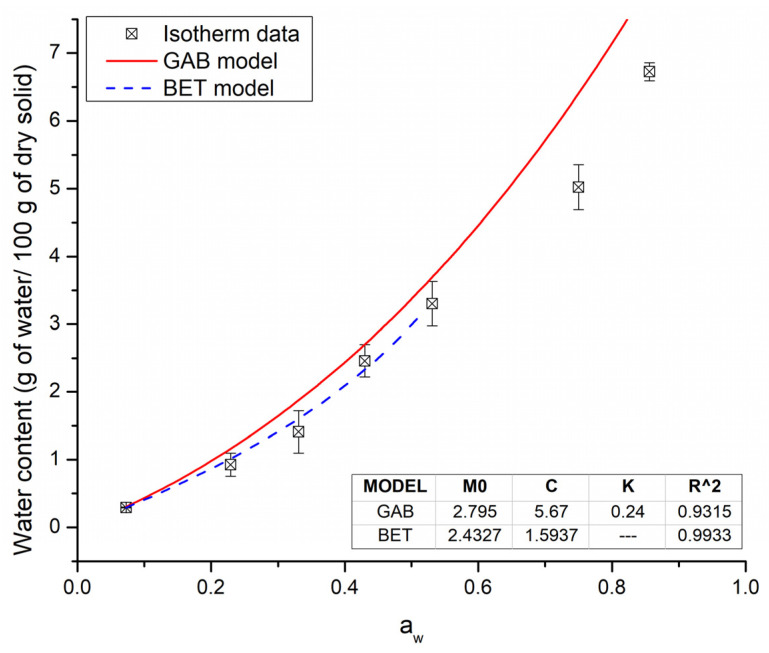
Water adsorption isotherm at 35 °C for the functional food blend MX-IN:25-75.

Sample MX-IN:25-75 was subjected to the adsorption of water in equilibrium at different conditions of humidity. Figure 2 shows the water adsorption isotherm at 35 °C for sample MX-IN:25-75. From the distinct adsorption conditions, the microenvironment created with K_2_SO_4_ produced the saturation of the powder and the subsequent collapse of the microstructure. This was observed as a continuous-phase material (not powdered), sticky, and with a remarkable yellow color. Because of this, the corresponding data for a_w_ (0.98) was not included in the plot nor considered for the physicochemical characterizations. 

The adsorbed water content varied in the range of 0.29–6.72 g of water per 100 g of dry sample in the complete interval of a_w_ (0.073–0.85). In Figure 2, the first observable characteristic is the shape of the isotherm, which presented a low moisture adsorption at low water activities and then a pronounced increase at medium and high a_w_ values. According to IUPAC, the shape of the isotherm was identified as the Flory-Huggins isotherm (type III). This isotherm accounts for a solvent or plasticizer above the glass transition temperature [36] and is typical of soluble, low-molecular-weight substances like sugars [37]. Several mathematical models for describing sorption isotherms were fitted to the experimental data. The evaluation of these models and their estimated parameters are reported in Figure S1 and Table S1 from the Supplementary Material. From the tested models, some did not fit the experimental data correctly, while others gave unrealistic results. Only those with consistent results are discussed herein. In the low-medium range of a_w_, the Brunauer-Emmett-Teller (BET) model was fitted. The BET monolayer value was 2.43 g of water per 100 g of dry sample, and the energy constant (C) related to the heat of sorption was 1.59. However, this model is limited to the linear range of water activity (0.073–0.531), and beyond this range, the BET model underestimates the predicted values. The Guggenheim-Anderson-de Boer (GAB) model has been used to fit isotherms across a wide range of a_w_ (0–0-9) and provides a more accurate description of sorption behavior for almost every food product [36]. The calculated GAB monolayer content (M_0_) was 2.795 g of water per 100 g of dry sample, and the model constants were 5.67 and 0.24, for C and K, respectively. The values of M_0_ calculated from the BET and GAB models were very similar and indicated the maximum amount of adsorbed solvent in the form of a monolayer of water molecules on the sample, which is considered the value at which the food product is the most stable [36]. Beyond this value, there are more water molecules available, acting as plasticizers, triggering unwanted microstructure changes, and acting as media for biological reactions such as bacterial growth. Therefore, to maintain the stability during storage of the functional food, it may be kept at a moisture condition lower than the value of the monolayer.

The C and K constants from the GAB model are related to the interaction energy between the first and further adsorbed layers of molecules, respectively. According to Lewicki [38], the estimated value of the monolayer will vary 15% when the values of the constants are within the following limits: 5.67 < C < ∞, and 0.24 < K < 1. The results obtained from the fit of the data indicated a good estimation of the monolayer value. 

### 3.3. Microstructural Analysis

**Figure 3:**
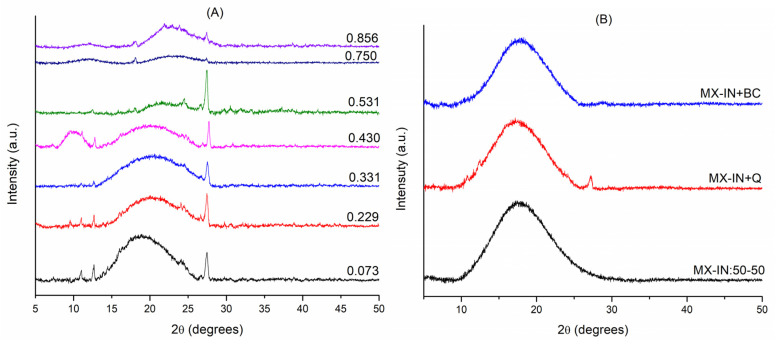
XRD diffractograms: (**A**) functional food blend MX-IN:25-75 at the different a_w_; and (**B**) blank samples.

The samples stored at different moisture conditions were analyzed by X-ray diffraction to observe possible changes in the microstructure. Figure 3A shows the X-ray diffractograms of the functional food blend MX-IN:25-75 at the different water activities. Microstructural behavior was similar in samples with an a_w_ of 0.073–0.331, with a broad peak near an angle of 18° and a relatively low intensity diffraction peak at 26.5°. These properties indicated that the microstructure remained amorphous across the entire range of water conditions. For a_w_ values of 0.43 and 0.531, the intensity of the broad peak decreased and small diffraction peaks appeared, suggesting that the amorphous microstructure is crystallizing due to moisture adsorption. At high a_w_ values of 0.73 and 0.856, the diffractograms showed a completely collapsed microstructure, which can be understood as a mixture of crystallized material and material in the rubbery state. 

According to previously reported studies, the amorphous microstructure of low molecular weight MXs is maintained with the adsorption of moisture, and only the change from a glassy solid into a rubbery state is observed a_w_ greater than 0.750 [39]. Meanwhile, with water adsorption, INs present a microstructural transition from amorphous to crystalline, observed as the appearance of diffraction peaks on a broad peak at a_w_ value of 0.32. The intensity of the diffraction peaks increases in a_w_ increments until the broad peak completely disappears [29]. Certainly, the blending of these two carbohydrate polymers with contrasting behaviors upon water adsorption causes the microstructural changes reported herein.

On the other hand, the diffraction peak observed at 26.5° suggested the crystallization of some of the active ingredients, such as quercetin or the microorganism *B. cluassi*. To verify the above, three blank samples containing only the mixture of carrier agents (MX-IN:50-50), the mixture plus quercetin (MX-IN+Q), and the mixture plus *B. claussi* (MX-IN+BC) were prepared and characterized by X-rays. Figure 3B shows the diffractograms for the blank samples. In all of these, the same broad peak around 18° was observed. The MX-IN+Q sample presented a diffraction peak at 26.5°, while the MX-IN+BC sample did not show any diffraction peak but only a small, broad peak around 28°. 

Clearly, these analyses help to understand the microstructural behavior of the functional food of the MX-IN:25-75 blend during storage at the different moisture conditions tested.

### 3.4. Morphological Characterization

**Figure 4:**
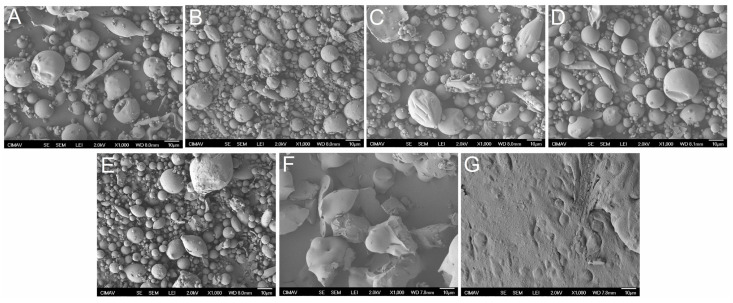
SEM micrographs of the functional food blend MX-IN:25-75 at the different a_w_ values: (**A**) 0.073, (**B**) 0.229, (**C**) 0.331, (**D**) 0.43, (**E**) 0.531, (**F**) 0.75, and (**G**) 0.856.

The result of storing information on the morphology of functional food particles was studied by SEM. Figure 4 depicts representative 1000X SEM micrographs of the functional food blend MX-IN:25-75 in various moisture environments. At a_w_ values of 0.073–0.531, the powder particles presented a quasi-spherical morphology, with sizes ranging from 2–15 microns. Particles with smooth surfaces were observed; however, other particles with an irregular (wrinkled) surface were also present. Likewise, some particles with elongated shapes were also presented, which may correspond to microencapsulated *B. claussi* microorganisms. For a_w_ of 0.75, the powder showed a different morphology, similar to merged spherical particles, forming irregular-shape agglomerates with sizes greater than 10 μm. At a_w_ of 0.856, the analyzed sample was no longer visualized as a powder but as a continuous mass.

These observations evidenced the changes exerted by the adsorption of water at the microscopic level. The functional food retains powder characteristics up to a maximum a_w_ value of 0.531. The morphology of the functional food changed dramatically above this humidity level, and it behaved as a crystallized solid and sticky material. Clearly, this analysis is of great importance because it sets the water activity range where the morphology is preserved before the microstructural changes may affect the properties of the powder.

### 3.5. Thermal Analysis

**Figure 5:**
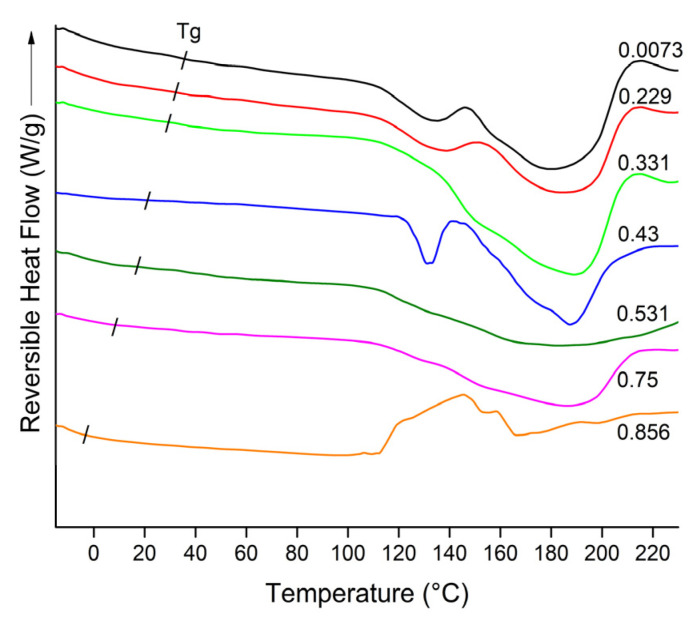
Reversible heat flow curves of the functional food blend MX-IN:25-75 at the different a_w_.

From the MDSC measurements, the glass transition temperature (Tg) of blend MX-IN:25-75 was determined after storage at different humidity conditions. Figure 5 shows the reversible heat curves at each a_w_. The determination of the Tg was performed graphically by identifying the onset of the reversible heat flow curve. The summary of Tg values is reported in Table 1. It is observed that the Tg tends to decrease with the increase of a_w_, and the variation of the Tg was in the range of 32.53 to −3.05 °C. This is a common behavior in sugar-rich systems, where the adsorbed water molecules act as plasticizers, decreasing the temperature to the point where the amorphous solid passes from a glassy to a rubbery state. Thus, it is preferable to keep the functional food in its glassy state because the microstructure of the solid remains rigid, preventing active ingredient degradation due to exposure to ambient atmospheric conditions.

Additionally, the Tg value indicates the maximum temperature at which the food can be stored before undergoing a microstructural change that may modify its composition. In this sense, samples with low moisture content (a_w_ of 0.0073–0.331) can be stored at temperatures close to 30 °C, while those with intermediate moisture content (a_w_ of 0.43 and 0.531) should be stored at temperatures below 20 °C, whereas samples with high moisture content (a_w_ of 0.75 and 0.856) should be kept at temperatures below 10 °C.

### 3.6. Functional Food Performance

**Figure 6:**
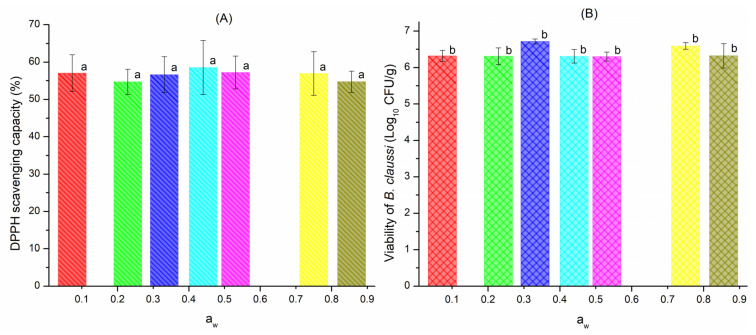
Functional performance of the blend MX-IN:25-75: (**A**) antioxidant activity of quercetin and (**B**) viability of *B. claussi* microorganisms. The lowercase letters indicate differences in the means at a significance value of 0.05.

The performance of blend MX-IN:25-75 as a functional food was tested by determining both the antioxidant activity of quercetin and the viability of *B. claussi* microorganisms. Figure 6 shows the AA and the viability of the blend MX-IN:25-75 subjected to different humidity conditions during storage at 35 °C.

Figure 6A shows that the powders presented antioxidant activity in the range of 54.6–58.5% of DPPH scavenging capacity. According to the ANOVA, the population means were not significantly different at the 0.05 level. By comparing the values reported herein against those reported in [10], the AA is the double of the former. They reported the AA for 1:1 ratio blends of MX-IN and compared them against samples prepared with pure MX or IN. They found that IN promotes the microencapsulation of quercetin, showing a synergistic effect in the blend. In the case of the blend reported here, it contains 75% of IN, which may favor the microencapsulation of quercetin and hence higher AA values.

Figure 6B shows the viability of *B. claussi* microorganisms at the different a_w_. The variation in viability was narrow, showing values between 6.3–6.7 Log_10_ CFU/g. In consequence, the ANOVA showed that the population means were not significantly different at the 0.05 level. This suggested that the blend MX-IN:25-75 was able to microencapsulate the microorganisms and keep them alive after the drying process and during storage at 35 °C under the different humidity conditions. On the other hand, the values found here were relatively lower than those reported in [10]. This can be explained in terms of the MX concentration, since MX favors the microencapsulation of the microorganism and its protecting effect is more pronounced than that of IN. This may be caused by the difficulty that microorganisms may have in metabolizing the long carbohydrate chains of IN [40]. Vázquez-Maldonado et al. compared IN and lactose as co-microencapsulating agents of resveratrol and *B. claussi*. They found that IN showed higher capacity for the conservation of resveratrol, while both materials showed similar viability values for encapsulating the microorganism [41]. Although the viability values reported herein were not very high, they were slightly above the minimum value recommended by the FAO/WHO (2003) to cause therapeutic benefits in humans. Therefore, the tested blend MX-IN:25-75 can be considered as functional food with probiotic properties.

In terms of storage stability, it is evident that the results are very promising, since the blend MX-IN:25-75 presented functional characteristics such as antioxidant activity and viability in the complete interval of water activities. This indicates that although the morphology of the particles may collapse at high humidity values, the active ingredients remain protected and active to convert the carbohydrate polymers into a functional food.

## 4. Discussion

### Equilibrium State Diagram of Functional Food Blend MX-IN:25-75

Commonly, two types of state diagrams are reported to estimate stability during storage. The first is based on determining the glass line, freezing curve, maximal freeze concentration, and solid mass fraction (Xs) [25]. For example, Wan et al. [42] determined for indica rice starch a Tg of −42.5 °C, an Xs of 0.71 g/g (wet basis), and a monolayer content of 7.43 g of water per 100 g of dry solids. Sablani et al. [43] determined for *basmati* rice a Tg of −11.6 °C, an Xs of 0.6, and a monolayer content value of 0.136 g of water per 100 g of dry solids. On the other hand, equilibrium state diagrams can be established by combining the water sorption isotherms and Tg for determining critical values for water content and water activity at a given storage temperature [44]. 

**Figure 7:**
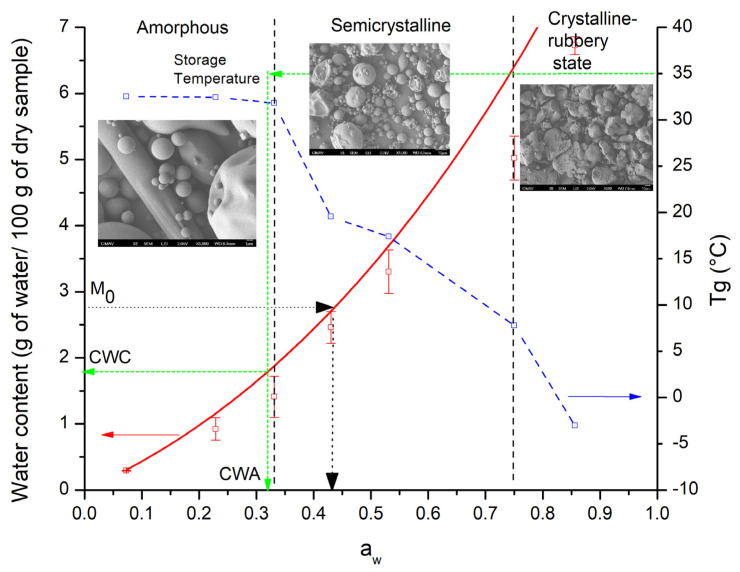
Equilibrium state diagram at 35 °C of functional food blend MX-IN:25-75.

Figure 7 shows the equilibrium state diagram constructed for the functional food blend MX-IN:25-75 from the results of the physicochemical characterization. One of the main features in the state diagram is the moisture adsorption isotherm at 35 °C (the red solid curve). Here is indicated the water content at the monolayer level (M_0_ of 2.79 g of water per 100 g of dry sample), corresponding to an a_w_ of 0.43. Moisture values greater than those of the monolayer will produce microstructural changes that can affect the behavior of the powder material. Thomsen et al. [45] constructed a state diagram for amorphous lactose to set the storage temperature and water activity at which lactose may remain in the glassy state. The depicted isotherm at 25 °C was employed as the borderline for separating the storage conditions for stable from unstable amorphous lactose. Fabra et al. [46] employed the concepts of a_w_, Tg, and the critical water content (CWC) to set the storage conditions of grapefruit. Because of the high water content of the fruit, it must be stored at freezing temperatures.

The Tg (blue dashed curve) shows a tendency to decrease with moisture adsorption. Three regions with similar glass transition temperatures were observed: (i) For moisture values lower than the monolayer level, the Tg showed values slightly higher than 30 °C. (ii) In the region of intermediate moisture values, Tg values were close to 19 °C. (iii) At high moisture contents, the Tg drops to values below 10 °C. According to Sá and Sereno [47], if the storage temperature equals the Tg, degradation of fresh fruits and vegetables may be prevented. Employing spray drying, Roos [48] constructed a state diagram employing the Tg and the mass fraction of lactose to establish the drying and storage temperatures of non-sticking powders from lactose-containing dairy liquids. In accordance with this, the powder must be stored at a lower temperature than the Tg of the main component. 

Additionally, from both the equilibrium state diagram and storage temperature (35 °C), the CWC and critical water activity (CWA) can be extrapolated. These values (indicated by the dashed green line) were a CWC of 1.77 g of water per 100 g of dry sample and a CWA of 0.32. Both values are lower than the corresponding values obtained from the moisture content of the monolayer. These results agree with those in the literature. For example, Shi et al. [49] reported lower CWC values for seafood meat (*Penaeus vannamei*) than those obtained from the monolayer. They also consider the CWC parameter to be of greater importance for the evaluation of the storage conditions of dehydrated food products. Vásquez et al. [50] emphasized the importance of CWC over M_0_, and stated that lyophilized blueberries must be stored at a_w_ less than 0.1 in order to preserve the glassy state of the matrix and avoid deteriorative changes in the amorphous components. Conversely, Sablani et al. [51] found constant CWA values of about 0.56 and CWC values of 0.11, 0.09, and 0.06 g of water per g of dry solid for abalone samples stored at 23, 40, and 60 °C, respectively. These CWC values were relatively higher than those determined from the BET monolayer content and may disturb the stability of the food product. Shi and collaborators [52] depicted an equilibrium moisture content diagram to evaluate the stability settings for storage of freeze-dried edible fungi (*Agaricus bisporus*) and reported a monolayer moisture content of 0.062 g of water per g of *A. bisporus* and a Tg of −27.2 °C. The addition of a carrying agent such as MX to freeze-dried seafood meat promoted an increment in the CWC from 0.151 to 0.258 and in a_w_ from 0.038 to 0.083 g of water per g of dry solids [49]. The joint use of concepts such as a_w_, CWC, and Tg in an equilibrium state diagram is of great importance to explain variations in time-dependent mechanical and flow properties, such as crystallization of amorphous sugars, non-enzymatic browning, and enzymatic reactions [44].

The vertical dashed lines indicate the water activities at which the microstructural changes occur. From here, three regions were identified: amorphous, semi-crystalline, and crystalline-rubbery states. This means that the microstructural change of this mixture is not abrupt but gradual, since it presents a transition state such as the semi-crystalline region. The value of the monolayer approximately matches the region where the microstructure transforms from the amorphous state to the semi-crystalline state. Roos [44] established that the effect of Tg on the mechanical properties results in rapid changes in viscosity and modulus, which cause the crystallization of amorphous solids. He identified three regions that depend on storing temperature and water activity or content. These regions were identified as: (i) a stability zone, which corresponds to a glassy state; (ii) a critical zone, where the glass transition occurs; and (iii) a mobility zone, where the flow occurs. According to Rahman [53], the microbial stability in food is affected by the moisture content, while the oxidation phenomenon is caused by the crystallization at a temperature higher than the Tg. 

A representative micrograph of the morphology of the powdered food particles is shown in each of the abovementioned regions. The behavior of the morphology corresponds with the microstructural fluctuations, resulting in spherical particles with smooth surfaces in the amorphous region. In the transition region, spherical particles with smooth surfaces were observed, as were particles with irregular shapes and contracted or collapsed surfaces. In the crystalline-rubbery state region, the morphology was completely irregular, with clusters of fused particles. According to Sahin et al. [53], the encapsulation efficiency can be affected by the presence of surface defects such as dents and heterogeneities since the active ingredients can escape through there. Al-Ghamdi et al. [54] used state diagrams to relate color change and browning with a_w_ and storage temperature of pumpkin powders. Both characteristics increased linearly with moisture content and at storage temperatures greater than 35 °C. Thanatuksorn, Kajiwara, and Suzuki [55] studied by SEM the structural alteration of fried wheat flour with various moisture contents. They observed that the porous structure reached its maximum value after 3 and 5 min of drying in samples containing 400 and 600 g of moisture per kg of sample, respectively. 

In addition to the information on phase and state changes provided by the microstructural and thermal characterizations, the morphological analysis provides information about the heterogeneities in the microstructure of the food, which is required for understanding the properties and kinetic behavior of amorphous food systems [44]. According to Buera et al. [56], among the characterization methods for studying the crystallization of amorphous sugars are DSC, XRD, microscopy, infrared, and Raman spectroscopies, whereas proton nuclear magnetic resonance (^1^H NMR) and dynamic mechanical analysis (DMA) have been employed as alternative techniques to determine Tg.

## 5. Conclusions

In this work, the use of a blend based on carbohydrate polymers as a matrix for a functional food with antioxidant and probiotic properties was proposed. The functional food was made up of 25% maltodextrin, 75% inulin, and the active ingredients quercetin and *Bacillus claussi*. The physicochemical characterization of the powders obtained by spray drying included moisture adsorption isotherms, X-ray diffraction, scanning electron microscopy, and thermal analysis. With the characterization results, an equilibrium state diagram for the storage stability of the functional food was constructed. The storage conditions were identified as a monolayer water content of 2.79 g of water per 100 g of dry sample, corresponding to an a_w_ of 0.43, and a maximum storage temperature slightly above 30 °C. The state diagram presented three microstructural regions identified as amorphous, semicrystalline, and crystalline-rubbery states. The morphology of particles varied in each of these regions, with spherical particles having smooth surfaces, a mixture of spherical and irregular particles having heterogeneous surfaces, and agglomerated and fused irregular-shaped particles being observed. The equilibrium state diagram was of great importance for identifying the storage conditions that promote the preservation of functional food properties and those where the collapse of the microstructure occurs.

The analysis of the functional properties of the blend showed antioxidant activities of about 50% of DPPH scavenging capacity and similar viability values of 6.5 Log_10_ CFU/g. These results demonstrated that the blend could be considered a functional food prepared by spray drying. 

## Figures and Tables

**Table 1:** Determined Tg values at different a_w_. The values represent the average of two measurements, and the standard deviation is indicated in brackets.

a_w_	Tg (°C)
0.0073	32.53 (1.3)
0.229	32.45 (0.9)
0.331	31.82 (1.1)
0.43	19.54 (0.6)
0.531	17.38 (1.8)
0.75	7.79 (0.4)
0.856	−3.05 (0.9)

## Data Availability

The data presented in this study are available on request from the corresponding author.

## Supplementary Materials

The following supporting information can be downloaded at: https://www.mdpi.com/article/10.3390/polym15020367/s1, Figure S1. Evaluation of the different sorption isotherm models; Table S1. Estimated parameters for the evaluated sorption models.
