# Supplementary material for: An Equilibrium State Diagram for Storage Stability and Conservation of Active Ingredients in a Functional Food Based on Polysaccharides Blends
Source: Polymers (Basel). 2023 Jan 10;15(2):367. doi: 10.3390/polym15020367 (PMC9860543; doi:10.3390/polym15020367)
Supplement: Supplementary file 1 [file polymers-15-00367-s001.zip › polymers-2126635-supplementary.pdf]

Supplementary Material

Figure S1 shows the evaluation of the different sorption isotherm models. The parameters of the models are presented in Table S1. Five models were evaluated to fit the experimental data. These models are known as: Brunauer-Emmett-Teller (BET), Guggenheim-Anderson-de Boer (GAB), Oswin, Smith, and Halsey [36].

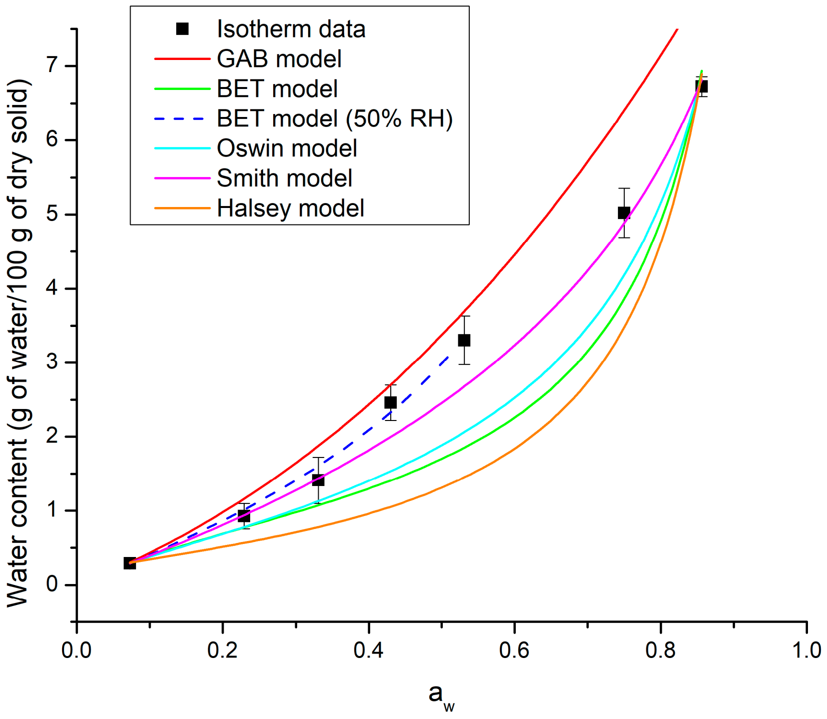

Figure S1. Evaluation of the different sorption isotherm models.

Table S1. Estimated parameters for the evaluated sorption models.

MODEL	EQUATION	PARAMETERS	R^2
BET	$M_W = \frac{M_0\ C\ a_w}{(1-a_w)(1+(C-1)a_w)}$	M_0= 1.03<br>C= 4.46	0.979
GAB	$M_W = \frac{M_0\ C\ K\ a_w}{(1-Ka_w)(1-Ka_w+CKa_w)}$	M_0= 2.79<br>C= 5.67<br>K= 0.24	0.954
Oswin	$M_W = C\left(\frac{a_w}{1-a_w}\right)^n$	C= 1.88<br>n= 0.72	0.985
Smith	$M_W = C_1 + C_2(1-a_w)$	C_1= 0.027<br>C_2= -3.5	0.997
Halsey	$M_W = \left(\frac{-C}{ln a_w}\right)^{\frac{1}{n}}$	C= 0.883<br>n= 0.900	0.963
BET (50% RH)	$M_W = \frac{M_0\ C\ a_w}{(1-a_w)(1+(C-1)a_w)}$	M_0= 2.43<br>C= 1.59	0.994
